# Epigenetic gene deregulation in cancer

**DOI:** 10.1054/bjoc.2000.1549

**Published:** 2000-12

**Authors:** K Malik, K W Brown

**Affiliations:** Cancer and Leukaemia in Childhood (CLIC) Research Unit, Department of Pathology and Microbiology, School of Medical Sciences, University of Bristol, University Walk, Bristol, BS8 1TD, UK

**Keywords:** epigenetic deregulation, tumour suppressor genes, genomic imprinting

## Abstract

A mini-review of the literature concerning epigenetic gene regulation in cancer. © 2000 Cancer Research Campaign http://www.bjcancer.com


*British Journal of Cancer* (2000) **83**(12), 1583–1588
© 2000 Cancer Research Campaign
doi: 10.1054/ bjoc.2000.1549, available online at http://www.idealibrary.com on IDEAL®

http://www.bjcancer.com

Mini-review

### Epigenetic gene deregulation in cancer

**K Malik and KW Brown**

Cancer and Leukaemia in Childhood (CLIC) Research Unit, Department of Pathology and Microbiology, School of Medical Sciences, University Walk, University of Bristol, Bristol BS8 1TD, UK

**Summary** A mini-review of the literature concerning epigenetic gene regulation in cancer. © 2000 Cancer Research Campaign http://www.bjcancer.com

**Keywords:** epigenetic deregulation; tumour suppressor genes; genomic imprinting

Over the last two decades, technical advances in molecular biology have led to a vast increase in our understanding of cancer. In particular, the discovery of two types of genes, proto-oncogenes and tumour suppressor genes (TSGs), have underpinned our expanding comprehension of the abnormalities that distinguish cancer cells from their normal counterparts. Broadly speaking, proto-oncogenes direct cells towards proliferation and prolonged survival, whereas tumour suppressor genes tend to inhibit, or keep in check, cell growth and survival. During normal development, tissue homeostasis requires a fine balance between these processes, whereas the equilibrium in tumour cells is disturbed, with the growth imperative taking over. Although proto-oncogenes and TSGs constitute only a small part of the cell's genetic repertoire, their pivotal role in cell propagation mark them as primary targets for mutations and deletions in cancer. Our ability to detect these changes at the molecular level has provided us with the vital blueprints for cancer genetics.

In addition to the catalogue of qualitative defects such as mutations and deletions which have been unravelled by the genetic dissection of cancers, subtle modifications of the nucleotide backbone leading to deregulation of gene expression have recently been emphasized as further distinguishing features of cancer cells. Specifically, these changes consist of variations of the DNA methylation patterns that overlay the primary structure of the genome, and, logically, the study of this field has been termed *epi*genetics, the prefix 'epi-' meaning 'upon', or 'in addition to'. As discussed below, DNA methylation is strongly associated with transcriptional repression and is linked with chromosomal architecture. The advent of epigenetics has therefore necessitated the conceptual refinement of the genetic blueprint, requiring a sense of genomic topography, with regions of the genome being segregated as transcriptionally active or inactive.

In order to assess the implications of epigenetic deregulation in cancer, this article will first summarize some biochemical aspects of DNA methylation. We will then consider examples of genes whose expression is altered in cancer cells and the possible consequences for the cell.

*Received 26 September 2000*
*Accepted 6 October 2000*

*Correspondence to:* K Malik. E-mail: k.t.a.malik@bris.ac.uk

### DNA methylation occurs at CpG islands

Mammalian cells are endowed with the possibility of a 'fifth base', namely 5-methylcytosine which can be formed and maintained enzymatically at any CpG dinucleotide in the genome. Sequence regions where there is a high density of CpG residues are termed CpG islands, and are loosely defined as being sequences of 200-plus base pairs with a G+C content of greater than 50% and a CpG/GpC ratio of > 0.6 (Gardiner-Garden and Frommer, 1987). These CpG islands are associated with gene promoters in approximately 50% of genes (Antequera and Bird, 1993) and are generally maintained in an unmethylated state, possibly via protection by transcription factors such as Sp1 (Brandeis et al, 1994). The notable exceptions for the purposes of this review are tumour-specific CpG island hypermethylation of TSGs, and imprinted genes which display parental-specific allelic gene expression in conjunction with differential methylation of alleles.

Three major cellular enzymic activities associated with DNA methylation have been characterized to date, including two subtypes of DNA methyltransferases, *DNMT1* and *DNMT3* (Okano et al, 1999; Robertson et al, 1999), and a DNA demethylase (Bhattacharya et al, 1999). DNMT1 is considered as a maintenance methyltransferase, this role being dramatically demonstrated in *DNMT1* knockout mice which die before birth, and in which genomically imprinted genes show inappropriate methylation in tandem with altered gene expression in the fetus (Li et al, 1993). This results in either complete loss of expression, or biallelic expression which is, of course, tantamount to overexpression. DNMT3 enzymes possess a de novo methylation activity (Okano et al, 1999) and their expression, together with that of *DNMT1* has been shown to be increased in some tumour cells (Robertson et al, 1999). Although tumour-specific elevation of *DNMTs* appeals as a causative step in cases of TSG hypermethylation observed in, for example, colorectal cancers (Issa et al, 1993), recent investigations along these lines have been equivocal, showing a lack of correlation between CpG island hypermethylation and *DNMT* overexpression (Eads et al, 1999). In fact a recent

**Table 1** Epigenetically altered genes in human cancers

| Silenced gene | Function | Tumour type | Reference |
|---|---|---|---|
| *VHL* | Promoter of angiogenesis | Clear cell renal | Herman et al, 1994 |
| *p16$^{INK4A}$* | Cyclin dependent kinase inhibitor | Many solid tumours, lymphomas | Herman et al, 1995 |
| *p15$^{INK4A}$* | CDK inhibitor | Haematological malignancies | Batova et al, 1997 |
| *Rb* | Cell cycle regulator | Retinoblastoma | Ohtani-Fujita et al, 1997 |
| *BRCA1* | Transcriptional regulator | Breast and ovarian | Estellar et al, 2000 |
| *E-cadherin (CDH1)* | Cell adhesion molecule | Gastric, breast and prostate | Grady et al, 2000 |
| *TIMP-3* | Matrix metalloproteinase inhibitor | Brain, colon and kidney | Bachman et al, 1999 |
| *hMLH1* | Mismatch repair gene | Colon and gastric | Kane et al, 1997 |
| *O6-MGMT* | DNA repair | Brain, colon lung and lymphoma | Estellar et al, 1999 |
| *Cyclooxygenase 2 (COX2)* | Prostaglandin metabolism | Colon | Toyota et al, 2000 |
| *Caspase 8 (CASP8)* | Apoptotic protease | Neuroblastoma | Teitz et al, 2000 |
| *14-3-3σ (stratifin)* | Cell cycle regulator | Breast | Ferguson et al, 2000 |
| *HOXA5* | Transcription factor | Breast | Raman et al, 2000 |
| *RASSF1A* | RAS signalling? | Lung | Dammann et al, 2000 |
| **Imprinted genes** | | | |
| *IGF2* | Growth factor | Wilms' tumour (WT) many others | Joyce and Schofield 1998 |
| *IGF2-AS* | Regulatory RNA? | WT | Okutsu et al, 2000 |
| *H19* | Regulatory RNA? | WT | Feinberg, 1999 |
| *CDKN1C/p57$^{KIP2}$* | CDK inhibitor | WT | Feinberg, 1999 |
| *BWR1A* | Apoptotic signal mediator? | WT, breast, lung | Feinberg, 1999 |
| *BWR1C* | Apoptotic signal mediator? | WT | Schwienbacher et al, 2000 |
| *WT1-AS* | Regulatory RNA? | WT | Malik et al, 2000 |
| *ARHI* | Growth regulator | Breast and ovarian | Xu et al, 2000 |
| *ZAC1* | Apoptotic regulator | Breast and ovarian | Kamiya et al, 2000 |
| *p73* | p53 homologue | Renal cell carcinoma and others | Mai et al, 1998 |

report has shown that methylation and silencing of TSGs is maintained in cancer cells in which the *DNMT1* gene has been completely disrupted by homologous recombination, although some satellite regions do show demethylation (Rhee et al, 2000). This suggests that regional specificities exist for DNMTs. Also, other as yet unidentified DNMTs may remain to be discovered, and perhaps the balance of cellular DNMT and DNA demethylase activity (of which little is known as yet) may be crucial in determining the epigenotype of the cancer cell.

### Mechanisms for methylation-mediated gene repression

The ability of the DNA methylation inhibitor 5-azacytidine to reactivate genes turned off by promoter methylation provides direct evidence that methylation of DNA can repress transcription (see for example Dammann et al, 2000). There are two main mechanisms by which nucleotide methylation is proposed to lead to transcriptional silencing of genes. Firstly, methylation of CpGs within transcription factor binding sites in promoters may block their binding and inhibit gene expression. For example, in retinoblastomas, binding-sites for trans-activating factors in the *RB* promoter were suggested to be inactivated by methylation (Ohtani-Fujita et al, 1997), and similarly a putative cAMP-responsive element binding-site (CREB) in the *BRCA1* promoter was compromised by methylation in breast and ovarian tumours (Mancini et al, 1998). The second repression mode uses the CpG methyl groups as tags that, via methyl binding domain (MBD) proteins such as MeCP2, recruit complexed histone deacetylases (HDACs). The targeted nucleosomal histone deacetylation mediated by HDACs leads to a condensed chromosomal architecture that is not conducive to the formation of transcription activating complexes at promoters. The association of MeCP2/HDAC with transcriptional repression suggests that methylation changes are potentially causative rather than merely a secondary consequence of tumorigenesis. Several MBD/HDAC complexes have been identified recently (Li, 1999), and these represent exciting candidates for modulators of various methylation signals. Interestingly, both pRB and BRCA1 proteins, whose expression has been shown to be silenced by hypermethylation, have also been found in complexes with chromatin remodelling activity (Brehm et al, 1998; Bochar et al, 2000). This raises the possibility that transcriptional deregulatory cascades might result from an initial genetic or epigenetic lesion.

### Silencing of TSGs by hypermethylation

Although methylation changes in cancer cells were recognized over a decade ago, it is only over the last 6 years that a detailed understanding of the phenomenon has emerged. The first part of Table 1 presents a cross-section of genes whose activity is silenced in various malignancies, including classic tumour suppressor genes known to be mutated in familial cancers, and subsequently shown to be epigenetically silenced in sporadic cancers. In the case of the *p16$^{INK4A}$* gene (Herman et al, 1995), the entire spectrum of gene disruption, genetic and epigenetic, is apparent in a wide variety of cancers, making it one of the most frequently inactivated tumour suppressor genes More recent examples of genes severely compromised in specific cancers include 14-3-3σ (stratifin), a facilitator of G2 arrest that is silenced by hypermethylation in over 90% of breast cancers (Ferguson et al, 2000), and caspase 8 (*CASP8*), a component of apoptotic proteolytic cascades that is silenced in approximately 60% of neuroblastomas (Teitz et al, 2000).

An obvious caveat to assessing hypermethylation changes is whether the epigenetic defects are secondary events, rather than the primary cellular insult in tumorigenesis. In colorectal cancers, various genes have been shown to be epigenetically silenced (see Table 1), and a CpG island methylator phenotype (CIMP) which predisposes cells to gene silencing has been described (Toyota et al, 1999). One possible route to selecting a hypermethylation phenotype has been suggested by Breivik and Gaudernack (1999) based

 *© 2000 Cancer Research Campaign*

on the formation of $O^6$-methylguanine adducts by bile acids in the proximal colon. These adducts may be inefficiently repaired by $O^6$-methylguanine-DNA methyltransferase ($O^6$-MGMT) due to epigenetic silencing of this gene (Estellar et al, 1999), and thereafter, cells bearing this mutation are locked in to an ineffectual cycle of attempted mismatch repair and ultimately death. Epigenetic silencing of the mismatch repair gene *hMLH* then allows cells to progress; thus natural selection forces a hypermethylator phenotype, facilitating the silencing of other genes in colorectal cancer. As this hypothesis illustrates, the initiating event in tumorigenesis might easily be plural in nature, and subsequent events, in view of the genes targeted, are likely to be important in tumorigenesis and also provide important markers for cancer. DNA repair genes silenced through promoter methylation, such as *$O^6$*-MGMT and *hMLH*, may therefore be envisaged as conducting elements between mutation and epimutation. Recent evidence for the causative nature of epigenetic lesions is available from studies of the gene encoding E-cadherin (*CDH1*) in hereditary diffuse gastric cancer (HDGC). Patients with germline mutations in *CDH1* and not exhibiting loss of the remaining allele were shown to inactivate the remaining allele by promoter hypermethylation (Grady et al, 2000), fulfilling Knudson's two-hit hypothesis with a primary genetic hit and a secondary epigenetic hit. Another variation of a compound epigenetic/genetic lesion possible in cancer was suggested by the work of Raman et al (2000). Although the mutation rate of *p53* is relatively low in sporadic breast cancer, levels of the protein are discernibly lower in breast tumour cells relative to normal epithelium. Lowered *p53* paralleled a decreased *HOXA5* expression, a transactivator of the *p53* promoter. The *HOXA5* promoter region was shown to be silenced by extensive methylation, and epigenetic silencing of the *HOXA5* gene can therefore alter *p53* activity and thereby compromise cellular defence against malignant transformation.

It remains to be elucidated as to whether other cancers can be subgrouped according to their epiphenotypes, and it will be of great interest to see whether, for example, *HOXA5* and 14-3-3σ hypermethylation in breast cancers occurs concurrently.

### Multiple expression modes regulated by DNA methylation

As discussed above, most gene-associated CpG islands are maintained in the unmethylated state, and it is therefore unsurprising that there are few reports of oncogene activation by hypomethylation. Imprinted genes are an exception to the general hypomethylated CpG island profile as they bear an epigenetic mark applied during gametogenesis, which results in the expression of only one parental allele (monoallelic expression). DNA methylation acts as an essential (though not necessarily the primary) epigenetic signal (Feil and Khosla, 1999), and *DNMT1* knockout mice show inappropriate methylation accompanied by either complete loss of expression, or biallelic expression, of imprinted genes (Li et al, 1993). Methylation of the parental alleles occurs differentially at specific sites (differentially methylated regions; DMRs), and can correlate with expression or non-expression of that allele. This distinction from TSG silencing by promoter hypermethylation is attributable to the existence of multiple CpG-rich regulatory elements and mechanisms of transcriptional control for imprinted genes. An example of a DNA regulatory region that acts as a positive and negative regulator of gene expression is the imprinting-control region (ICR) at the *IGF2-H19* locus. These genes display reciprocal allelic expression, *IGF2* from the paternal allele and *H19* from the maternal allele.

Methylation of a paternal allele DMR 5′ of the *H19* gene (the imprinting control region) inactivates a chromatin boundary between *IGF2* and *H19*, by blocking binding of the CTCF protein. This in turn allows the *IGF2* gene preferential access to a set of enhancers shared by the two genes, which leads to *IGF2* expression from the paternal allele (Reik and Murrell, 2000). Thus, whereas methylation of the paternal allele ICR inactivates *H19* expression, it facilitates *IGF2* expression, the ICR acting as a chromatin boundary element. Other epigenetic pathways for *IGF2* regulation which are not contingent on the ICR are also suggested by detailed methylation analysis of the *IGF2* region in Wilms' tumours (WTs) by sequencing of bisulphite-modified DNA (Sullivan et al, 1999). Using this method, which assesses methylation at all CpG dinucleotides by the selective conversion of unmethylated cytosines to uracil, with methylated cytosines remaining unaltered (Olek et al, 1996), a novel DMR was identified in the *IGF2* gene. Whereas the *IGF2* promoters and other CpG islands exhibited no methylation changes paralleling the imprinting status of samples, the new DMR displayed an absence of methylation in WTs in which *IGF2* imprinting became biallelic, implicating this region in tumour-specific deregulation.

### Genomic imprinting changes in cancer

One of the earliest links between imprinting and cancer came with the discovery that loss of heterozygosity (LOH) at chromosome 11p always involved the loss of the maternal allele in WTs. This preferential allele loss can be interpreted as being driven either by the necessity in tumours to lose a maternally expressed growth inhibitory gene, or to retain a paternally expressed growth factor (Reik and Surani, 1989). A dense cluster of imprinted genes has subsequently been uncovered at 11p15, containing both the paternally expressed growth factor insulin-like growth factor 2 (*IGF2*), and the maternally expressed growth inhibitory genes *H19* and *CDKN1C* (*$p57^{KIP2}$*) (Feinberg, 1999). This suggests that the loss of the maternal allele at 11p15 leads to the retention of expression of a growth-promoting gene, *IGF2*, and the loss of expression of growth inhibitory genes (*H19* and *CDKN1C*).

Examination of WTs not showing 11p15 LOH revealed consistent biallelic expression of *IGF2*; this has been termed 'relaxation of imprinting' (ROI) or 'loss of imprinting' (LOI) (Rainier et al, 1993). Biallelic expression of *IGF2* in WTs is associated with the loss of expression of the tightly linked *H19* gene, and with the hypermethylation of an associated ICR on the normally unmethylated maternal allele (Moulton et al, 1994; Reik and Murrell, 2000). The *IGF2* maternal allele that is normally silent becomes active, thereby leading to an increase in *IGF2*-gene dosage (Hastie, 1994). In addition to the somatic epigenetic changes in WT, altered imprinting of the 11p15 region is involved in a tumour susceptibility syndrome, the Beckwith–Wiedemann syndrome (BWS). BWS is a fetal overgrowth syndrome, exhibiting organomegaly and increased risk of childhood cancer, especially WT. Familial cases of BWS map to 11p15, and some sporadic cases have chromosomal abnormalities involving the same region. Each of these genetic abnormalities exhibits parent-of-origin effects: preferential maternal transmission of inherited BWS, and in sporadic cases uniparental disomy, paternally derived chromosome duplications, and maternally derived chromosome translocations (Maher and Reik, 2000). This suggested that imprinting defects might underlie

© 2000 Cancer Research Campaign

BWS, and indeed, constitutional biallelic expression of *IGF2* was found in several BWS patients, implicating relaxed imprinting of this fetal mitogen as the primary cause of the disease (Weksberg et al, 1993). The increased dosage effect of uniparental disomy in BWS is functionally comparable to tumours showing LOH of chromosome 11p15 in that LOH is accompanied by duplication of the remaining (paternal, non-imprinted) copy of the gene (Hastie, 1994).

Since the original reports, loss of *IGF2* imprinting has been found in a large number of different types of both childhood and adult cancers, including breast and hepatocellular cancers. While *IGF2* cannot be regarded as a classic proto-oncogene as no activating somatic mutations have been detected in cancer, its overexpression may serve to give a proliferative advantage to tumour cells in many different tissues (Joyce and Schofield, 1998). In a mouse model of multistage carcinogenesis where tumours are produced by SV40 T-antigen expression in the pancreas, loss of *IGF2* imprinting is an early event in tumour development (Christofori et al, 1995). This is supported by the demonstration of LOI of *IGF2* in the normal colonic mucosa of patients with colorectal cancer (Cui et al, 1998). Epigenetic lesions in *H19* are also detectable in normal kidney tissue adjacent to WTs, and in premalignant lesions (nephrogenic rests) (Moulton et al, 1994; Okamoto et al, 1997). Taken together, this suggests that imprinting changes can be an early event in tumorigenesis.

Interestingly, Cui et al (1998) also demonstrate a strong association of *IGF2* loss of imprinting with colorectal cancers with microsatellite instability, which is linked to epigenetic silencing of the mismatch repair gene *hMLH* (Kane et al, 1997). Silencing of *hMLH* does not, however, occur in normal colonic mucosa suggesting that sequential epigenetic defects contribute to the development of the microsatellite instability tumour phenotype.

Other notable imprinted genes on chromosome 11p15 include *BWR1A* which shows low levels of somatic mutation in a variety of cancers (Feinberg, 1999) and *BWR1C* (Schwienbacher et al, 2000). *BWR1C* is expressed from the maternal allele in fetal kidney, but not in adult kidney or other adult tissues. Expression of the gene is specifically down-regulated in WTs irrespective of their LOH status by an as yet unidentified mechanism, with no variation in promoter methylation being apparent. As WTs arise from the developing kidney by the abnormal proliferation of renal stem cells (metanephric blastema), they bear many oncofetal characteristics, such as high expression of *IGF2* and *WT1* (Hastie, 1994). Therefore it is of particular interest that *BWR1C*, which has homology to pro-apoptotic proteins, is turned off in a tumour-specific manner. Some imprinted genes such as the mouse insulin-like growth factor 2 receptor (*Igf2r*) have been shown to be allelically silenced through oppositely imprinted antisense transcripts (Wutz et al, 1997), and it will be of interest to examine whether *BWR1C* is regulated by an antisense transcript. Alternatively, *cis*-acting elements such as those at the *IGF2-H19* locus may be epigenetically altered near the *BWR1C* gene.

In addition to the 11p15 gene cluster, several other imprinted loci implicated in diverse malignancies have been reported recently (see Table 1). We have recently demonstrated relaxation of imprinting in WT of an antisense transcript (*WT1-AS*) from the *WT1* gene on chromosome 11p13, which correlated with hypomethylation of an intronic DMR. In contrast to genetic changes at 11p13, which occur in approximately 20% of WTs, we found this epigenetic modification in 80% of WTs with no LOH at 11p13 (Malik et al, 2000). The *WT1* antisense transcript is imprinted in normal kidney, whereas the sense RNA is not, although *WT1* sense transcripts show polymorphic and mosaic imprinting in other tissues (Hastie, 1994). Therefore, *WT1-AS* does not act as an allelic silencer in kidney, and although the function of *WT1-AS* is unclear, we have shown that it can modulate WT1 protein levels in vitro and that it is co-expressed with *WT1* sense RNA and protein in vivo (Moorwood et al, 1998). The antisense RNA may act to stabilize the sense transcript in a manner analogous to the translocation-specific *bcl2*-IgH antisense transcript found in human follicular lymphoma cells and suggested to up-regulate *bcl-2* expression (Capaccioli et al, 1996). As inappropriate *WT1* expression has been associated with increased tumorigenic potential (Menke et al, 1996), we have hypothesized that *WT1-AS/WT1* may be oncogenic when expressed inappropriately (Malik et al, 2000). However, it remains to be determined when ROI takes place, and it will clearly be of great interest to investigate epigenetic events in nephrogenic trests. Recently, coordinate overexpression of an imprinted antisense *IGF2* transcript (*IGF2AS/PEG8*) and *IGF2* has also been shown in WT (Okutsu et al, 2000), although *IGF2AS* overexpression is not as a result of LOI. These studies emphasize the need for a fuller assessment of antisense RNA regulation and its role in normal development and tumorigenesis.

Two novel imprinted loci on the short arm of chromosome 1 have recently been described. These include the *NOEY2/ARHI* tumour suppressor gene at 1p31 (Yu et al, 1999), a maternally imprinted gene undergoing deletion of the non-imprinted (expressing) allele in over 40% of breast and ovarian cancers. Transgenic mice overexpressing the *ARHI* gene exhibit decreased growth and development (Xu et al, 2000). In contrast, the *p73* gene, a *p53* homologue, at 1p36 shows loss of imprinting leading to biallelic expression in renal cell carcinoma, implying oncogenic activity (Mai et al, 1998).

Finally, the *ZAC1/LOT1* gene was identified via its loss on transformation (hence LOT) in an ovarian tumour model (Abdollahi et al, 1997). It is located on chromosome 6q24–25, a region frequently deleted in many solid tumours, and the gene product has been shown to inhibit growth and induce apoptosis, suggesting TSG activity (Varrault et al, 1998). The gene has recently been shown to be maternally imprinted (Kamiya et al, 2000), but tumour-specific epigenetic changes remain to be characterized.

### Conclusion

The epigenetic dissection of normal and tumour cell genomes represents perhaps the most immediately fecund post-genome initiative. However, although targets for silencing by promoter hypermethylation may be relatively easy to assess, it is clear from our discussion that we are only beginning to understand more complex regulatory elements, molecules and pathways, such as chromatin boundary elements and antisense/non-coding RNAs. By employing a combinatorial approach examining both gene expression and DNA methylation profiling, it should become possible to obtain a detailed comprehension of the cancer cell's transcriptome and its cognate regulatory apparatus. Our understanding of epigenetics and its associated marker, DNA methylation, provides a crucial cornerstone for future explorations. In contrast to classical mutations, epigenetic changes are potentially reversible by pharmaceutical intervention. As there is increasing evidence that epigenetic lesions are causative in nature, the

 *© 2000 Cancer Research Campaign*

benefits of epigenetics research are potentially high, including methods for cancer prognosis, diagnosis, and ultimately treatment. Modulators of general methylation (5-azacytidine) and histone deacetylation (trichostatin, butyrate) may provide useful templates and adducts for more specific cancer therapies.

### ACKNOWLEDGEMENTS

We greatly appreciate the work of the many researchers in the field whose publications could not be cited due to editorial constraints. We also wish to thank Professor Christos Paraskeva for helpful comments. Work in our laboratory was funded by the Cancer and Leukaemia in Childhood (CLIC) charity and a Medical Research Council (UK) studentship.

### REFERENCES

Abdollahi A, Godwin AK, Miller PD, Getts LA, Schultz, DC, Taguchi T, Testa, JR and Hamilton TC (1997) Identification of a gene containing zinc-finger motifs based on lost expression in malignantly transformed rat ovarian surface epithelial cells. *Cancer Res* **57**: 2029–2034

Antequera F and Bird A (1993) Number of CpG islands and genes in human and mouse. *Proc Natl Acad Sci USA* **90**: 11995–11999

Bachman KE, Herman JG, Corn PG, Merlo A, Costello JF, Cavenee WK, Baylin SB and Graff JR (1999) Methylation-associated silencing of the tissue inhibitor of metalloproteinase-3 gene suggest a suppressor role in kidney, brain, and other human cancers. *Cancer Res* **59**: 798–802

Batova A, Diccianni MB, Yu JC, Nobori T, Link MP, Pullen J and Yu AL (1997) Frequent and selective methylation of p15 and deletion of both p15 and p16 in T-cell acute lymphoblastic leukemia. *Cancer Res* **57**: 832–836

Bhattacharya SK, Ramchandani S, Cervoni N and Szyf M (1999) A mammalian protein with specific demethylase activity for mCpG DNA. *Nature* **397**: 579–583

Bochar DA, Wang L, Beniya H, Kinev A, Xue Y, Lane WS, Wang W, Kashanchi F and Shiekhattar R (2000) BRCA1 is associated with a human SWI/SNF-related complex: linking chromatin remodeling to breast cancer. *Cell* **102**: 257–265

Brandeis M, Frank D, Keshet I, Siegfried Z, Mendelsohn M, Nemes A, Temper V, Razin A and Cedar H (1994) Sp1 elements protect a CpG island from de novo methylation. *Nature* **371**: 435–438

Brehm A, Miska EA, McCance DJ, Reid JL, Bannister AG and Kouzarides T (1998) Retinoblastoma protein recruits histone deacetylase to repress transcription. *Nature* **391**: 597–601

Breivik J and Gaudernack G (1999) Carcinogenesis and natural selection: a new perspective to the genetics and epigenetics of colorectal cancer. *Adv Cancer Res* **76**: 187–212

Capaccioli S, Quattrone A, Schiavone N, Calastertti A, Copreni E, Bevilacqua A, Canti G, Gong L, Morelli S and Nicolin A (1996) A bcl-2/IgH antisense transcript deregulates bcl-2 gene expression in human follicular lymphoma t(14;18) cell lines. *Oncogene* **13**: 105–115

Christofori G, Naik P and Hanahan D (1995) Deregulation of both imprinted and expressed alleles of the insulin-like growth factor 2 gene during beta-cell tumorigenesis. *Nat Genet* **10**: 196–201

Cui H, Horon IL, Ohlsson R, Hamilton SR and Feinberg AP (1998) Loss of imprinting in normal tissue of colorectal cancer patients with microsatellite instability. *Nat Med* **4**: 1276–1280

Dammann R, Li C, Yoon JH, Chin PL, Bates S and Pfeifer GP (2000) Epigenetic inactivation of a RAS association domain family protein from the lung tumour suppressor locus 3p21.3. *Nat Genet* **25**: 315–319

Eads CA, Danenberg KD, Kawakami K, Saltz LB, Danenberg PV and Laird PW (1999) CpG island hypermethylation in human colorectal tumors is not associated with DNA methyltransferase overexpression. *Cancer Res* **59**: 2302–2306

Estellar M, Hamilton SR, Burger PC, Baylin SB and Herman JG (1999) Inactivation of the DNA repair gene $O^6$-methylguanine-DNA methyltransferase by promoter hypermethylation is a common event in primary human neoplasia. *Cancer Res* **59**: 793–797

Estellar M, Silva JM, Dominguez G, Bonilla F, Matias-Guiu X, Lerma E, Bussaglia E, Prat J, Harkes IC, Repasky EA, Gabrielson E, Schutte M, Baylin SB and Herman JG (2000) Promoter hypermethylation and BRCA1 inactivation in sporadic breast and ovarian tumors. *J Natl Cancer Inst* **92**: 564–569

Feil R and Khosla S (1999) Genomic imprinting in mammals: An interplay between chromatin and DNA methylation? *Trends Genet* **15**: 431–435

Feinberg AP (1999) Imprinting of a genomic domain of 11p15 and loss of imprinting in cancer: An introduction. *Cancer Res* **59**: 1743s–1746s

Ferguson AT, Evron E, Umbircht CB, Pandita TK, Chan TA, Hermeking H, Marks JR, Lambers AR, Futreal PA, Stampfer MR and Sukumar S (2000) High frequency of hypermethylation at the 14–3–3 σ locus leads to gene silencing in breast cancer. *Proc Natl Acad Sci USA* **97**: 6049–6054

Gardiner-Garden M and Frommer M (1987) CpG islands in vertebrate genomes. *J Mol Biol* **196**: 261–282

Grady WM, Willis J, Guilford PJ, Dunbier AK, Toro TT, Lynch H, Wiesner G, Ferguson K, Eng C, Park J-G, Kim S-J and Markowitz S (2000) Methylation of the *CDH1* promoter as the second genetic hit in hereditary diffuse gastric cancer *Nat Genet* **26**: 16–17

Hastie, ND (1994) The genetics of Wilms' tumor – a case of disrupted development. *Ann Rev Genet* **28**: 523–558

Herman JG, Latif F, Weng Y, Lerman MI, Zbar B, Liu S, Samid D, Duan DS, Gnarra JR and Linehan WM (1994) Silencing of the VHL tumor-suppressor gene by DNA methylation in renal carcinoma. *Proc Natl Acad Sci USA* **91**: 9700–9704

Herman JG, Merlo A, Mao L, Lapidus RG, Issa JP, Davidson NE, Sidransky D and Baylin SB (1995) Inactivation of the CDKN2/p16/MTS1 gene is frequently associated with aberrant DNA methylation in all common human cancers. *Cancer Res* **55**: 4525–4530

Issa JP, Vertino PM, Wu J, Sazawal S, Celano P, Nelkin BD, Hamilton SR and Baylin SB (1993) Increased cytosine DNA-methyltransferase activity during colon cancer progression. *J Natl Cancer Inst* **85**: 1235–1240

Joyce JA and Schofield PN (1998) Genomic imprinting and cancer. *J Clin Pathol Mol Pathol* **51**: 185–190

Kamiya M, Judson H, Okazaki Y, Kusakabe M, Muramatsu M, Takada S, Takagi N, Arima T, Wake N, Kamimura K, Satomura K, Hermann R, Bonthron DT and Hayashizaki Y (2000) The cell cycle control gene ZAC/PLAGL1 is imprinted – a strong candidate gene for transient neonatal diabetes. *Hum Mol Genet* **9**: 453–460

Kane MF, Loda M, Gadia GM, Lipman J, Mishra R, Goldman H, Jessup JM and Kolodner R (1997) Methylation of the *hMLH1* promoter correlates with lack of expression of hMLH1 in sporadic colon tumors and mismatch repair-defective human tumour cell lines. *Cancer Res* **57**: 808–811

Li E (1999) The mojo of methylation. *Nature* **23**: 5–6

Li E, Beard C and Jaenisch R (1993) Role for DNA methylation in genomic imprinting. *Nature* **366**: 362–365

Maher ER and Reik W (2000) Beckwith-Wiedemann syndrome: Imprinting in clusters revisited. *J Clin Invest* **105**: 247–252

Mai M, Qian C, Yokomizo A, Tindall DJ, Bostwick D, Polychronakos C, Smith DI and Liu W (1998) Loss of imprinting and allele switching of p73 in renal cell carcinoma. *Oncogene* **17**: 1739–1741

Malik K, Salpekar A, Hancock A, Moorwood K, Jackson S, Charles A and Brown KW (2000) Identification of differential methylation of the WT1 antisense regulatory region and relaxation of imprinting in Wilms' tumor. *Cancer Res* **60**: 2356–2360

Mancini DN, Rodenhiser DI, Ainsworth PJ, O'Malley FP, Singh SM, Xing W and Archer TK (1998) CpG methylation within the 5′ regulatory region of the BRCA1 gene is tumor specific and includes a putative CREB binding site. *Oncogene* **16**: 1161–1160

Menke AL Riteco N van Ham, RCA de Bruyne C Rauscher III FJ van der Eb AJ and Jochemsen AG (1996) Wilms' tumor I splice variants have opposite effects on the tumorigenicity of adenovirus-transformed baby-rat kidney cells. *Oncogene* **12**: 537–546

Moorwood K, Charles AK, Salpekar A, Wallace JI, Brown KW, and Malik K (1998) Antisense WT1 transcription parallels sense mRNA and protein expression in fetal kidney and can elevate protein levels *in vitro*. *J Pathol* **185**: 352–359

Moulton T, Crenshaw T, Hao Y, Moosikasuwan J, Lin N, Dembitzer F, Hensle T, Weiss L, McMorrow L, Loew T, Kraus W, Gerald W and Tycko B (1994) Epigenetic lesions at the H19 locus in Wilms' tumour patients. *Nat Genet* **7**: 440–447

Ohtani-Fujita N, Dryja TP, Rapaport JM, Fujita T, Matsumura S, Ozasa K, Watanabe Y, Hayashi K, Maeda K, Kinoshita S, Matsumura T, Ohnishi Y, Hotta Y, Takahashi R, Kato MV, Ishizaki K, Sasaki MS, Horsthemke B, Minoda K and Sakai T (1997) Hypermethylation in the retinoblastoma gene is associated with unilateral, sporadic retinoblastoma. *Cancer Genet Cytogenet* **98**: 43–49

Okamoto K, Morison IM, Taniguchi T and Reeve AE (1997) Epigenetic changes at the insulin-like growth factor II/H19 locus in developing kidney is an early event in Wilms tumorigenesis. *Proc Natl Acad Sci USA* **94**: 5367–5371

© 2000 Cancer Research Campaign

Okano M, Bell DW, Haber DA and Li E (1999) DNA methyltransferases Dnmt3a and Dnmt3b are essential for de novo methylation and mammalian development. *Cell* **99**: 247–257

Okutsu T, Kuroiwa Y, Kagitani F, Kai M, Aisaka K, Tsutsumi O, Kaneko Y, Yokomori K, Surani MA, Kohda T, Kaneko-Ishino T and Ishino F (2000) Expression and imprinting status of human PEG8/IGF2AS, a paternally expressed antisense transcript from the IGF2 locus, in Wilms' tumors. *J Biochem* **127**: 475–483

Olek A, Oswald J and Walter J (1996) A modified and improved method for bisulphite based cytosine methylation analysis. *Nucelic Acids Res* **24**: 5064–5066.

Rainier S, Johnson LA, Dobry CJ, Ping AJ, Grundy PE and Feinberg AP (1993) Relaxation of imprinted genes in human cancer. *Nature* **362**: 747–749

Raman V, Martensen SA, Reisman D, Evron E, Odenwald WF, Jaffee E, Marks J and Sukumar S (2000) Compromised HOXA5 function can limit p53 expression in human breast tumours. *Nature* **405**: 974–978

Reik W and Murrell A (2000) Silence across the border. *Nature* **405**: 408–409

Reik W and Surani A (1989) Genomic imprinting and embryonal tumours. *Nature* **338**: 112–113

Rhee I, Jair K-W, Chiu Yen R-W, Lengauer C, Herman JG, Kinzler KW, Vogelstein B, Baylin SB and Schuebel KE (2000) CpG methylation is maintained in human cancer cells lacking DNMT1. *Nature* **404**: 1003–1007

Robertson KD, Uzvolgyi E, Liang G, Talmadge C, Sumegi J, Gonzales FA and Jones PA (1999) The human DNA methyltransferases (DNMTs) 1, 3a and 3b: coordinate mRNA expression in normal tissues and overexpression in tumors. *Nucleic Acids Res* **27**: 2291–2298

Schwienbacher C, Angioni A, Scelfo R, Veronese A, Calin GA, Massazza G, Hatada I, Barbanti-Brodano G and Negrini M (2000) Abnormal RNA expression of 11p 15 imprinted genes and kidney developmental genes in Wilms' tumor. *Cancer Res* **60**: 1521–1525

Sullivan MJ, Taniguchi T, Jhee A, Kerr N and Reeve AE (1999) Relaxation of IGF2 imprinting in Wilms tumours associated with specific changes in IGF2 methylation. *Oncogene* **18**: 7527–7534

Teitz T, Wei T, Valentine MB, Vanin EF, Grenet J, Valentine VA, Behm FG, Look At, Lahti JM and Kidd VJ (2000) Caspase 8 is deleted or silenced preferentially in childhood neuroblastomas with amplification of MYCN. *Nat Med* **6**: 529–535

Toyota M, Ahuja N, Ohe-Toyota M, Herman JG, Baylin SB and Issa JP (1999) CpG island methylator phenotype in colorectal cancer. *Proc Natl Acad Sci USA* **96**: 8681–8686

Toyota M, Shen L, Ohe-Toyota M, Hamilton SR, Sinicrope FA and Issa JJ (2000) Aberrant methylation of the cyclooxygenase 2 CpG island in colorectal tumours. *Cancer Res* **60**: 4044–4048

Varrault A, Ciani E, Apiou F, Bilanges B, Hoffmann A, Pantaloni C, Bockaert J, Spengler D and Journot L (1998) hZAC encodes a zinc finger protein with antiproliferative properties and maps to a chromosomal region frequently lost in cancer. *Proc Natl Acad Sci USA* **95**: 8835–8840

Weksberg R, Shen DR, Fei YL, Song QL and Squire J (1993) Disruption of insulin-like growth factor 2 imprinting in Beckwith-Wiedemann syndrome. *Nat Genet* **5**: 143–150

Wutz A, Smrzka OW, Schweifer N, Schellander K, Wagner EF and Barlow DP (1997) Imprinted expression of the Igf2r gene depends on an intronic CpG island. *Nature* **389**: 745–749

Yu Y, Xu F, Peng H, Fang X, Zhao S, Li Y, Cuevas B, Kuo W-L, Gray JW, Siciliano M, Mills GB and Bast RC Jr (1999) *NOEY2 (ARHI)*, an imprinted putative tumor supressor gene in ovarian and breast carcinomas. *Proc Natl Acad Sci USA* **96**: 214–219

Xu F, Xia W, Luo RZ, Peng H, Zhao S, Dai J, Long Y, Zou L Le W, Liu J, Parlow AF, Hung M, Bast RC and Yu Y (2000) The human ARHI tumor suppressor gene inhibits lactation and growth in transgenic mice. *Cancer Res* **60**: 4913–4920

*© 2000 Cancer Research Campaign*

